# A New Performance-Based Test for Assessing Chloride-Induced Reinforcement Corrosion Resistance of Geopolymer Mortars

**DOI:** 10.3390/ma17215162

**Published:** 2024-10-23

**Authors:** Kazuo Ichimiya, Rieru Yamamoto, Ko Ikeda, Quang Dieu Nguyen, Arnaud Castel

**Affiliations:** 1 Department of Civil and Environmental Engineering, National Institute of Technology, Oita College, Oita 870-0152, Japan; ichimiya@oita-ct.ac.jp; 2 Environmental & Social Solutions Business Unit, Kyushu System Department, NEC Networks & System Integration Corporation, Fukuoka 810-0001, Japan; yamamoto.rieru@nesic.com; 3 Graduate School of Science and Engineering, Yamaguchi University, 2-16-1 Tokiwadai, Ube 755-8611, Japan; k-ikeda@yamaguchi-u.ac.jp; 4 School of Civil and Environmental Engineering, University of Technology Sydney (UTS), Sydney 2007, Australia; quangdieu.nguyen@uts.edu.au

**Keywords:** geopolymer, steel corrosion, chloride, performance-based test, fly ash, ground granulated blast-furnace slag

## Abstract

The widespread adoption of geopolymer concretes in the industry has been slow, mainly due to concerns over their long-term performance and durability. One of the main causes of concrete structures’ deterioration is chloride-induced corrosion of the reinforcement. The reinforcement corrosion process in concrete is composed of two main stages: the initiation phase, which is the amount of time required for chloride ions to reach the reinforcement, and the propagation phase, which is the active phase of corrosion. The inherent complexities associated with the properties of precursors and type of activators, and with the multi-physics processes, in which different transfer mechanisms (moisture, chloride, oxygen, and charge transfer) are involved and interact with each other, have been a major obstacle to predicting the durability of reinforced alkali-activated concretes in chloride environments. Alternatively, the durability of alkali-activated concretes can be assessed through testing. However, the performance-based tests that are currently available, such as the rapid chloride permeability test, the migration test or the bulk diffusion test, are only focusing on the initiation phase of the corrosion process. As a result, existing testing protocols do not capture every aspect of the material performance, which could potentially lead to misleading conclusions, particularly when involving an electrical potential to reduce the testing time. In this paper, a new performance-based test is proposed for assessing the performance of alkali-activated concretes in chloride environments, accounting for both the initiation and propagation phases of the corrosion process. The test is designed to be simple and to be completed within a reasonable time without involving any electrical potential.

## 1. Introduction

Geopolymer (GP) concrete, a low-embodied-carbon alternative to Ordinary Portland Cement (OPC)-based concrete, has been at the forefront of recent academic research. While these materials have shown great potential for various construction applications, their commercial adoption has been relatively slow, mainly due to concerns over their long-term performance and durability. The main factors hindering the mass production of GP binders can be identified as follows: (1) the properties of the final product are highly sensitive to the properties of precursors, type of activators, the water/solid ratios, and curing age/regimes; (2) compared to OPC-based materials in which the main variable affecting the properties of the final product is the water/cement ratio, the design of alkali-activated and GP binders involves understanding a dynamic and interdependent set of variables. This is a major obstacle for general practitioners and small concrete suppliers; (3) the long-term performance and durability of these binders require further investigation, as they are comparatively new construction materials compared to OPC-based binders.

One of the main causes of concrete structures’ deterioration is chloride-induced corrosion of the reinforcement. The reinforcement corrosion process in concrete is composed of two main stages: the initiation phase, which is the required time for aggressive agents (e.g., chloride ions) to reach the reinforcement and depassivate it, and the propagation phase, which is the active phase of corrosion [1]. During the initiation phase of the chloride-induced corrosion process, the moisture transport properties of concrete define the rate of moisture adsorption and, consequently, the penetration of chloride ions through a convective process during wetting/drying cycles in non-saturated concrete. Once the concrete is saturated, however, chloride diffusivity is the main controlling factor. When enough chloride ions reach the interface between concrete and reinforcement, the passive layer around the bars starts to deteriorate and corrosion begins. Not all the penetrated chloride ions contribute to the depassivation process, as a percentage of them will be physically/chemically bound to the matrix. The amount of chloride that leads to the breakdown of the passive layer and the onset of corrosion is called the chloride threshold, and it is highly dependent on the chemical composition of the binder. During the propagation phase of corrosion, moisture adsorption and desorption play significant roles, as the electrical resistivity of concrete depends on its moisture content. Electrochemical parameters, such as the corrosion potential and polarization resistance, are other parameters that define the kinetics of corrosion during the propagation phase. Finally, a lack of oxygen, which depends on the pore structure, can decrease the rate of corrosion as the corrosion process becomes concentration-controlled. Although chloride-induced reinforcement corrosion processes have been the subject of much OPC-based concrete research in the past, the amount of literature for new and alternative binders such as GP binders is relatively small.

With regard to chloride diffusion in GP-type binders, the requirement for calcium in the matrix to form a finer gel structure to reduce the rate of chloride penetration is suggested in a number of previous studies, based on the observed superior performance of alkali-activated slag binders, or blended fly ash (FA) and slag binders, compared with GP-type or even OPC binders in chloride-contaminated environments [2,3,4,5]. Noushini et al. [6,7] found that a minimum of 50% ground granulated blast-furnace slag (GGBS) in FA-GGBS blended precursor systems is required to achieve acceptable resistance against chloride diffusion. Mixes with less than 25% GGBS performed poorly. Extremely low-chloride diffusion coefficients were observed for mixes with more than 75% GGBS.

Chloride threshold values (CTVs) for GP materials were recently reviewed by Tahri et al. [8]. Criado et al. [9] found that steel corrosion was initiated for chloride contents around 0.4 wt.% of binder, depending on the activator. However, Monticelli et al. [10] observed CTVs of around 1–1.7 wt.% of binder for alkali-activated FA mortars, which was significantly higher than those observed for OPC specimens (~0.5 wt.% of binder). Babaee and Castel [11] obtained a CTV of 0.19 for alkali-activated mortars with 75% slag and 25% FA contents, 3%Na_2_O (wt.% binder mass) and 0.69 for 75% FA and 25% slag contents, 8% Na_2_O which were in line with the recommendations for OPC-based binders [12]. In addition, it was noticed that CTVs of alkali-activated mortars (AAMs) decreased markedly with the increase in slag content, regardless of the alkali content or modulus ratio [11], which was resulted from less developed passive film in these binders that can be broken down in much easier ways. According to Mangat et al. [13], the threshold Cl^−^/OH^−^ value for pitting corrosion initiation in the slag-based alkali-activated concrete ranged between 2.1 and 2.8 compared with 1.08 for the control OPC concrete.

In the propagation phase, alkali-activated GGBS concrete showed higher corrosion current densities than OPC concrete due to a reducing environment around the steel surface in alkali-activated GGBS concrete, which was caused by a high sulfide concentration in the pore solution [13]. It was also reported that the corrosion resistance of alkali-activated GGBS concretes increases with the increase in the molarity of the alkali activator, at a constant liquid to binder ratio. Ma et al. [4] suggested 1.5 as an optimum modulus for the activator for improving the chloride transport and the corrosion resistance. Further, Sadangi and Pradhan [14] reported more negative half-cell potential and higher corrosion current density of steel reinforcement by increasing the molarity of the NaOH solution in GP concretes made with FA and GGBS with a proportion of 70/30. However, mostly less negative half-cell potential and lower corrosion currents were detected with a rise in the molarity of the NaOH solution in 100% FA-based GP concrete. Gunasekara et al. [15] investigated different FA-based GP concretes. The results also showed lower corrosion rates of steel bars in FA-based GP specimens compared to the Portland cement-based concrete. Babaee and Castel [16] reported that reinforced low-calcium FA-based GP concrete samples exhibit polarization resistance values comparable to those of OPC-based corroding systems.

Overall, the inherent complexities associated with the properties of precursors and type of activators, and with the multi-physics processes, in which different transfer mechanisms (moisture, chloride, oxygen, and charge transfer) are involved and interact with each other, have been a major obstacle to predicting the durability of reinforced alkali-activated concretes in chloride environments. Alternatively, the durability of alkali-activated concretes can be assessed through testing. However, the performance-based tests that are currently available, such as the rapid chloride permeability test, the migration test, and the bulk diffusion test, only focus on the initiation phase of the corrosion process. As a result, existing testing protocols do not capture every aspect of the material’s performance, which could potentially lead to misleading conclusions, particularly when involving an electrical potential to reduce the testing duration. In this paper, a new performance-based test is proposed for assessing the performance of alkali-activated concretes in chloride environments accounting for both initiation and propagation phases of the corrosion process. The test is designed to be simple and to be completed within a reasonable time without involving any electrical potential.

## 2. Outline of Tests

The mortar testing program included 7 days of standard compressive strength and a standard chloride ion permeability test (JSCE-G571 [17]), a new method for assessing the corrosion of steel bars through the accurate quantification of a bar’s rusted area using an image analysis method. The shrinkage and the expansion of the mortar specimens were also monitored over the whole period of the wetting/drying cycle exposure [18].

### 2.1. Geopolymer Mortar Materials and Mix Design

Table 1 and Table 2 show the materials and mix design used for the GP mortars. FA and GGBS were used as aluminosilicate precursors. The ratio of GGBS volume to total powder volume (hereinafter referred to as the GGBS replacement ratio) was 10%, 20%, and 30% (hereinafter referred to as BS10, BS20, and BS30). A mixture of sodium water glass, caustic soda, and tap water was used as the alkali solution. The alkali solution concentrations (A/W: alkali component/water) were 0.095, 0.126, and 0.187. Mixed silica sand was used as fine aggregate. 

### 2.2. Curing Method

**Figure 1:**
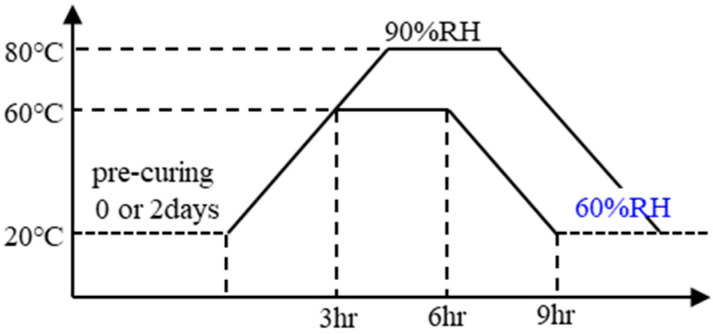
Curing conditions.

Figure 1 shows the different curing conditions. The specimens were cured with steam in a programmable constant-temperature/humidity apparatus at a maximum temperature of 60 °C or 80 °C, maintained for 3 h and a relative humidity (RH) of 90% or higher. Before heat curing, specimens were stored in their molds for either 0 or 2 days at 20 °C; this was referred to as the pre-curing period. Specimens were demolded after 1 day (in case of 0 pre-curing days) or 3 days (2 pre-curing days). When the heat curing procedure was complete, they were placed in a constant-temperature/humidity chamber (20 °C, 60%RH) for 7 days before being tested. The compressive test was performed on day 7 in order to shorten the total experimental period. The compressive strength test results provided are the average values of three samples.

### 2.3. Compressive Strength Test

The compressive strength test was conducted on the mortar prims (40 × 40 × 160 mm) in accordance with JIS R 5201 [19] and ISO 679 [20] after 7 days. The compressive strength test results provided are the average values obtained for three samples.

### 2.4. Chloride Ion Permeability Test

The mixture used in this test was Mix4-Mix6 (A/W = 0.126). The test was conducted in accordance with the Japan Society of Civil Engineers standard “Test method for effective diffusion coefficient due to migration of chloride ions in concrete” (JSCE-G571) [17].

**Figure 2:**
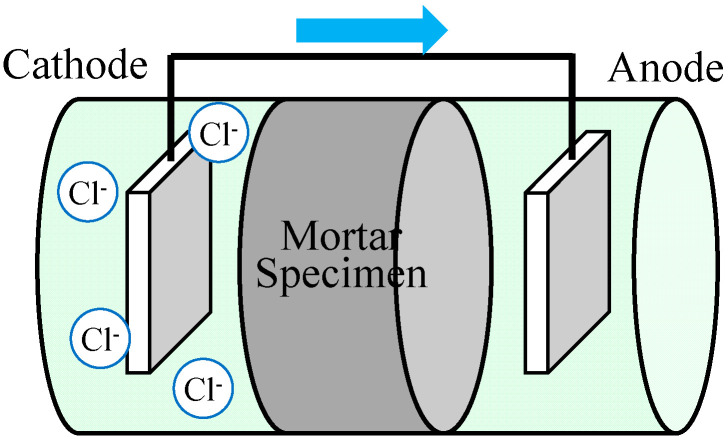
Schematic diagram of electrophoresis test apparatus.

Figure 2 shows a schematic diagram of the electrophoresis apparatus used for the chloride ion permeability test. The test principle is based on the movement of negatively charged chloride ions from the cathode side to the anode side of the apparatus, driven by a potential gradient. Mortar cylinders of 100 × 200 mm dia. were used as specimens, cut to 50 mm thickness. Surfaces other than the cut surface were coated with epoxy resin and vacuum-absorbed after resin hardening. The specimens were then set in an electrophoresis cell apparatus. Next, 0.5 mol/L NaCl solution was added to the cathode side and 0.3 mol/L NaOH solution was added to the anode side, and a DC voltage of 15 V was applied between the electrodes by a DC stabilizing power supply.

The chloride ion concentration was measured by potentiometric titration. The chloride content was calculated as follows:

(1) $$Cl(\%)=\frac{3.545\times \left(V_{1}-V_{2}\right)\times N}{W}$$

where *V*_1_ is the volume (mL) of AgNO_3_ solution used for sample titration, *V*_2_ is the volume (mL) of AgNO_3_ solution used for blank titration, *N* is the exact normality of AgNO_3_ solution, and *W* is the sample mass (g).

The chloride ion diffusion coefficient (DCL) was calculated by applying the Nernst–Planck equation using the flux of chloride ions (JCLs) under steady-state conditions, as presented in Equation (2). The DCL of OPC was taken from the literature as a comparison:

(2) $$J_{i}=-D_{i}\times \left(\frac{dC_{i}}{dx}+C_{i}\frac{d\ln\gamma_{i}}{dx}+Z_{i}C_{i}\frac{F}{RT}\cdot \frac{dE}{dx}\right)$$

where *J_i_* is the unidirectional flux of species *i* (mol/m^2^/s); *D_i_* is the diffusion coefficient of species *i* in the solution (m^2^/s); C is the concentration of species *i* (mol/m^3^); *x* is the distance (m); *γ* is the activity coefficient; *z_i_* is the electrical charge of species *i*; *F* is the Faraday constant (96,485); *R* is the gas constant (8.3145); *T* is the temperature (°K); and *E* is the electrical potential (V). The electrophoresis test results reported are the average values of three samples.

### 2.5. Corrosion of Steel Bars

The curing conditions are as shown in Figure 1. The rebars are 16 mm in diameter and 105 mm in length. The reason for using cylindrical specimens is that the distance between the surface of the specimen and the surface of the rebar is kept constant, and the degree of corrosion can be evaluated based on the area of rust over the whole steel surface. It is also easy to remove the rebars from the specimen by splitting them. Immersion time was 3 days, and drying time was 1 day. As with the splitting tensile test, the cylindrical specimen was placed horizontally into the testing machine and split into two parts by applying compressive loads from above and below.

**Figure 3:**
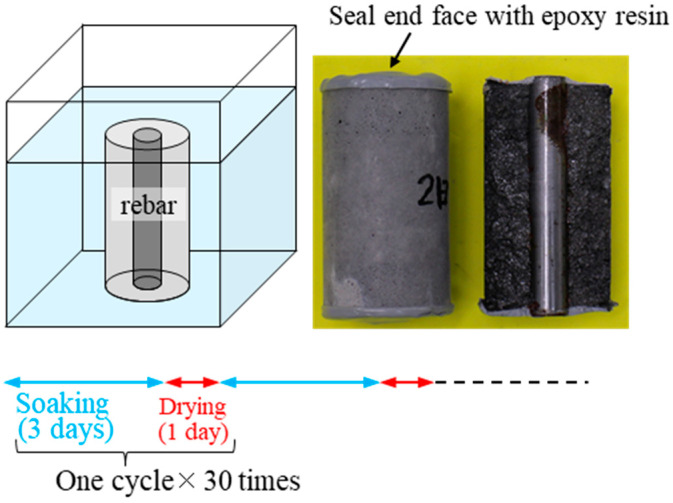
Rebar corrosion test involving immersion in salt water.

Mortar specimens (50 × 100 mm cylinders) with polished steel bars placed inside were immersed in salt water (0.5 mol/L NaCl solution) at 7 days of age, as shown in Figure 3, to investigate the relationship between the age of immersion and corrosion of the steel bars. In order to accelerate the corrosion of the steel bars, the test included up to 30 cycles of wetting and drying, with each cycle consisting of 3 days of immersion and 1 day of drying. The tests were conducted in a constant temperature and humidity chamber (20 °C, 60%RH).

**Figure 4:**
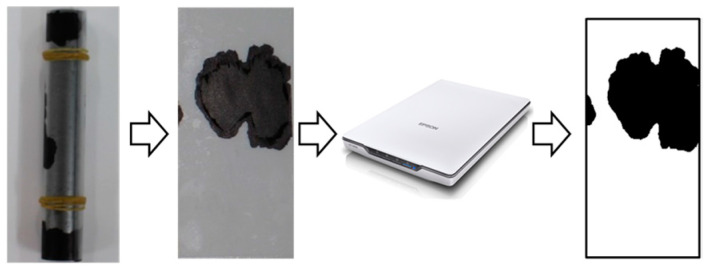
Rust area quantification by image processing.

Following the salt water immersion protocol, the specimens were split and the polished rebar was removed. Figure 4 shows the procedure for quantification of the rust area. The rusted area was digitized using a flatbed scanner and the total area of rust was measured using image analysis software.

For each mortar mix design, several samples were tested but only one was broken to assess the rust area ratio of the rebar at each testing age.

### 2.6. Length and Mass Change

**Figure 5:**
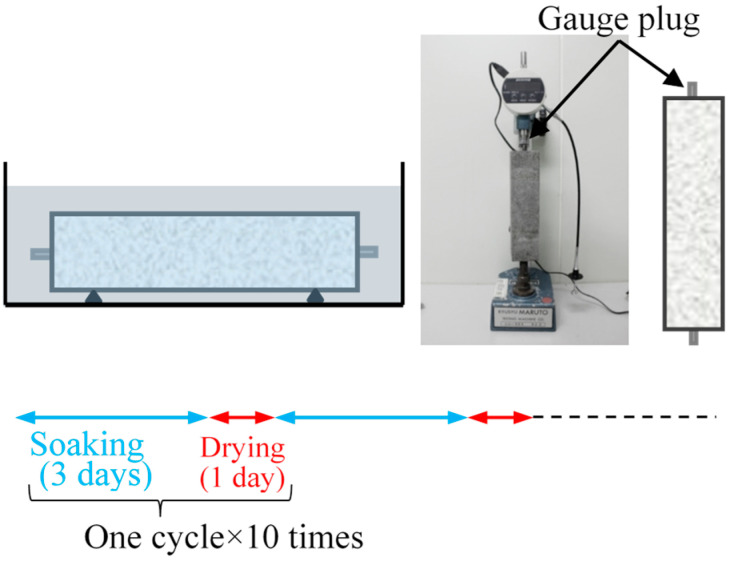
Measurement of the length change due to repeated drying and wetting cycles.

The length change test of mortar was conducted in accordance with JIS A-1129-3 “Method of measuring length change of mortar and concrete—Part 3: Dial gauge method” [21]. No rebar was used in this test. Drying/wetting cycles were performed for 10 cycles (40 days) using the method shown in Figure 5.

As a result of the rebar corrosion test, it was found that some specimens had cracks on their surfaces. Based on the expectation that the cracks were caused by shrinkage and expansion during the repeated wetting/drying cycles, a wetting/drying cyclic test was conducted using a plain mortar square prism specimen (40 × 40 × 160 mm). Figure 5 shows a schematic diagram of the wetting/drying cyclic test. In a constant-temperature and -humidity chamber (20 °C, 60%RH), the mortar was immersed in tap water for 3 days and air-dried for 1 day for 10 cycles, and the changes in the length and mass of the mortar after wetting and drying were monitored (Figure 5).

## 3. Results and Discussion

### 3.1. Compressive Strength

**Figure 6:**
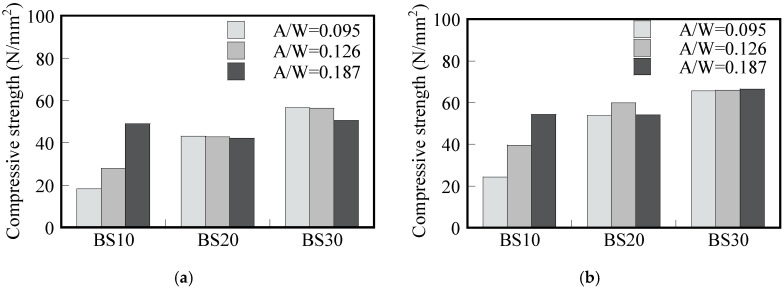

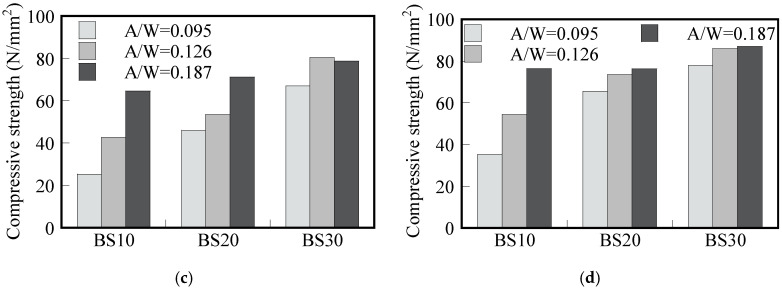
Effect of GGBS replacement ratio on 7-day compressive strength versus A/W ratio: (**a**) 60 °C and 0 days of pre-curing; (**b**) 60 °C and 2 days of pre-curing; (**c**) 80 °C and 0 days of pre-curing; (**d**) 80 °C and 2 days of pre-curing.

Figure 6 shows the 7-day compressive strength of the mortar after 0 and 2 days of pre-curing, respectively. A higher curing temperature of 80 °C (Figure 6a,b) produced higher compressive strength compared with specimens with the same GGBS replacement and the A/W ratio cured at 60 °C (Figure 6c,d). The compressive strength of specimens without pre-curing (Figure 6a,c) show the same trend as those that underwent 2 days of pre-curing, but the strength of specimens that underwent 2 days of pre-curing is 28% higher than that of those that underwent 0 days of pre-curing. This indicates the benefit of implementing 2 days of pre-curing to improve the compressive strength of GP.

Bakharev, Kani, and Allahverdi reported that longer periods of pre-curing performed at room temperature prior to heat curing could improve the geopolymerization and strength development when mainly FA was used in the binder [22,23]. For BS10, the compressive strength also increased when the A/W ratio increased for all pre-curing durations and curing temperatures. However, the A/W ratio seemed to have no significant influence for both BS20 and BS30 mortars, except for 80 °C and 0 days of pre-curing. This indicates that increasing the activator concentration only leads to a significant compressive strength increase when the GGBS content in the mix composition is 10% or lower. In general, the compressive strength values were also comparable those obtained in a previous study investigating the mechanical properties of low-calcium FA-based GP concrete at a wide range of heat-curing temperatures [24].

### 3.2. Chloride Ion Permeability

**Figure 7:**
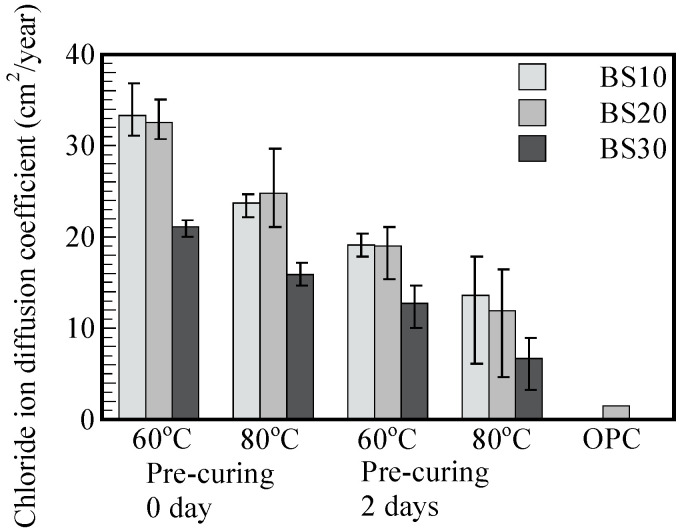
Chloride ion diffusion coefficients versus steam curing temperature, number of pre-curing days, and GGBS replacement rate (in the case of A/W = 0.126).

Figure 7 shows the diffusion coefficient of chloride ion (DCL) for each experimental condition for an A/W ratio of 0.126. The DCL of GP mortars is larger than that of OPC mortar. As mentioned above, the data for OPC mortar report differences of 1.5 cm^2^/year based on the existing literature [25]. The diffusion coefficient for BS30 is 50–64% higher than that for BS10 and BS20. The increase in GGBS replacement content from 10% to 30% contributed to the reduction in the DCL values in all GP mortars for both curing temperatures (60 °C and 80 °C) and pre-curing conditions (0 and 2 days).

**Figure 8:**
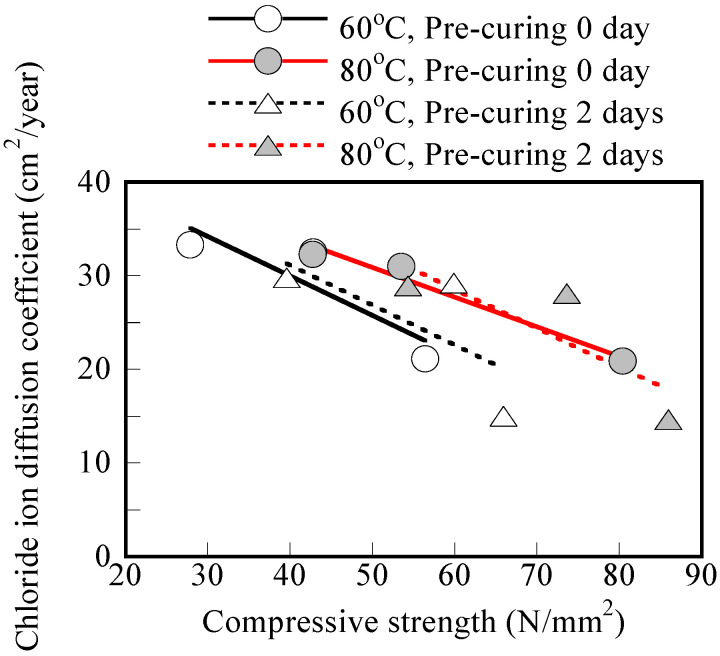
Relationship between chloride ion diffusion coefficient and 7-day compressive strength for different curing conditions (in the case of A/W = 0.126).

The decrease in chloride ion diffusion coefficient when replacing FA with GGBS in the binder compositions was reported in previous studies [6,7,11]. The DCL values of the GP mortar cured at 80 °C were lower than those of mortars cured at 60 °C, which is consistent with the higher compressive strength results in Section 3.1. Figure 8 shows a good correlation between 7 days of compressive strength and the chloride ion diffusion coefficient if the curing temperature and pre-curing duration are kept the same. According to the results shown in Figure 8, for an A/W ratio of 0.126, the effect of the number of pre-curing days seems as significant as the effect of the GGBS replacement rate. However, increasing the steam curing temperature seems to influence the DCL reduction the most. To be specific, specimens cured at 80 °C exhibit lower chloride ion diffusion coefficients ranging from 43.8% to 72.5% compared to the coefficients of specimens cured at 60 °C with same GGBS replacement and pre-curing conditions.

However, considering all of the test results together, the correlation between compressive strength and chloride ion diffusion coefficient is quite poor. For example, for a compressive strength of about 50 MPa, the chloride ion diffusion coefficient can range from 25 to 31 cm^2^/year, suggesting that compressive strength is not a good indicator of GP concrete/mortar durability in chloride environments.

### 3.3. Corrosion of Steel Bars

1. Effects of GGBS replacement rate

**Figure 9:**
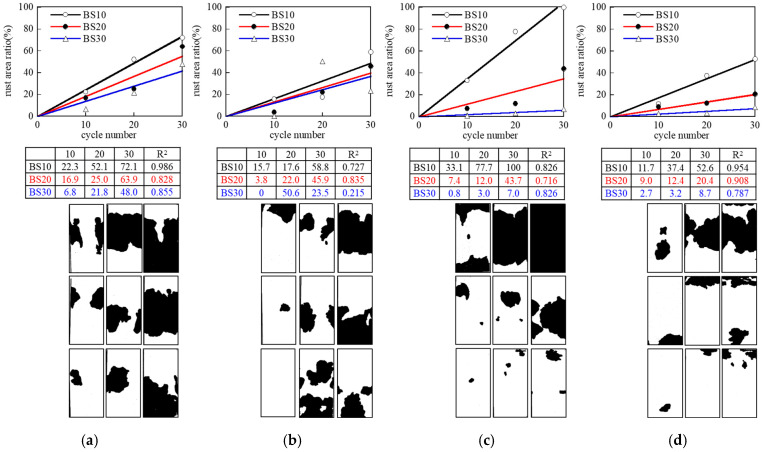
Rusting on surface (effect of GGBS replacement rate, number of days of pre-curing, and steam curing temperature in the case of A/W = 0.126): (**a**) 60 °C and 0 days of pre-curing; (**b**) 60 °C and 2 days of pre-curing; (**c**) 80 °C and 0 days of pre-curing; (**d**) 80 °C and 2 days of pre-curing.

Figure 9 shows the rusting of the rebar for up to 30 cycles and the relationship between the corrosion area ratio and the number of cycles. The rusting is highly correlated with the results of compressive strength (Figure 6) and the chloride ion diffusion coefficient (Figure 7 and Figure 8) and is likely to be the result of the densification of the mortar microstructure. On the other hand, it is not possible to completely eliminate rusting simply by adjusting the GGBS replacement ratio. More and comprehensive measurements that take other factors into consideration, such as the number of days of pre-curing, as described below, are necessary.

Figure 9 shows a strong correlation between the number of drying/wetting cycles and the corrosion area ratio for most experimental conditions. Only a few cases show a smaller corrosion area ratio after 30 cycles compared to 20 cycles. As the test is destructive, a different specimen is broken at each testing stage. This rare trend is certainly due to some variability in the mortar properties of the same mix design, also potentially leading to different steel/concrete interface conditions depending on the compaction applied. The main purpose of the test remains to identify geopolymer mortars that do not cause extensive rusting for at least 30 cycles.

2. Effects of number of days of pre-curing

Rusting was suppressed more in the case with 2 days of pre-curing than in the case that underwent 0 days of pre-curing in Figure 9. As with the GGBS replacement rate described above, the correlation between compressive strength and the chloride ion diffusion coefficient suggests that fewer/smaller voids formed during the 2-day pre-curing period. The strength-increasing effect of pre-curing has been reported in the past [22,23].

Figure 6 shows that the compressive strength of both formulations increased by 10–42% (27% on average) with the 2-day pre-curing period, with the rate of increase being greater with smaller GGBS replacement rates (BS10: 10–42%, BS20: 30%, BS30: 22%). In the FA-GGBS GP, N-A-S-H is mainly produced from FA and C-A-S-H from GGBS. The N-A-S-H is a tetrahedral 3D mesh structure nucleated by Al and Si ions leached from FA. It is possible that a denser GP matrix was formed by steam curing after sufficient dissolution. In addition, N-A-S-H has high adhesion to metal. For example, when GP mortar is placed into metal molds, it may adhere so strongly to the metal that demolding may be difficult under some experimental conditions. The same phenomenon was observed in this experiment. As shown in the photo on the right side of Figure 3, when the rebar was removed, the rebar and GP mortar adhered strongly to each other after 2 days of pre-curing. Therefore, it can be concluded that the number of days of pre-curing affects the formation of matrix gels (N-A-S-H, C-A-S-H), leading to a better rebar corrosion resistance after 2 days of pre-curing compared to no pre-curing.

Adhesion between the matrix and the rebar can be observed on the cross section of the cut specimen. The adhesion between GP and metal is an interesting phenomenon that is frequently observed. We believe that the mix proportions and curing conditions that allow the two to adhere strongly also contribute greatly to preventing the rebar from rusting, and this will be a topic for future studies.

3. Effects of steam curing temperature

The chloride ion diffusion coefficients shown in Figure 8 show little change with steam curing temperature. Consistent with the diffusion coefficient values, the results depicted in Figure 9 also show an insignificant difference in rebar corrosion between curing temperatures of 60 °C and 80 °C.

4. Effects of alkali solution concentration (A/W)

**Figure 10:**
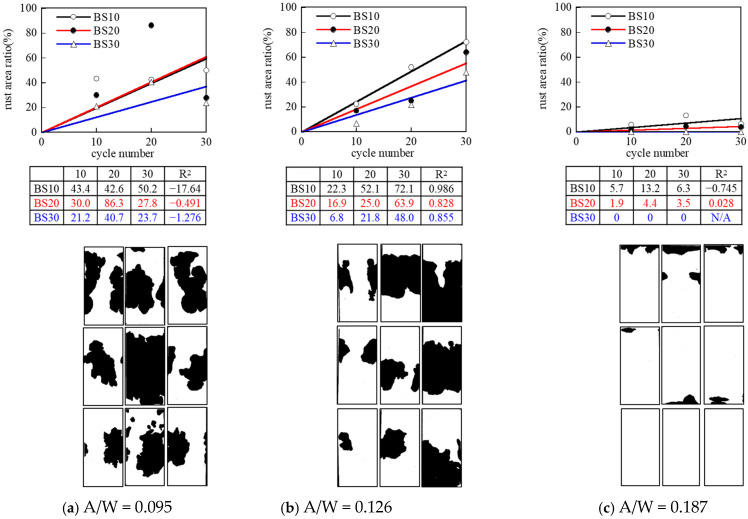
Rusting on rebar surface of influence of A/W (60 °C, 0 days of pre-curing).

Figure 10 shows the rusting condition for each A/W. Overall, rusting tends to be reduced as the A/W ratio increases. In particular, when A/W = 0.187, no rusting occurred in BS20 and BS30, indicating that the increase in alkali solution concentration (A/W) could decrease the corrosiveness of rebars.

Ma et al. also indicated that increasing the % Na_2_O (i.e., activator concentration) in mixture composition resulted in a reduction in the corrosion rate of the steel bars due to a low chloride diffusion coefficient [4]. On the other hand, the rusting in BS10 was caused by peeling between the epoxy resin applied to the edge of the specimen and the base metal, and if the sealing effect was sufficient, the rusting would not have occurred as in BS20 and BS30. In the repeated wetting/drying immersion test in salt water, it is desirable to use a test method that allows evaluation with as few wetting/drying cycles as possible to avoid delamination between the sealing material and the base metal at the specimen end face, as well as to shorten the test period.

5. Correlation with compressive strength and chloride ions permeability

**Figure 11:**
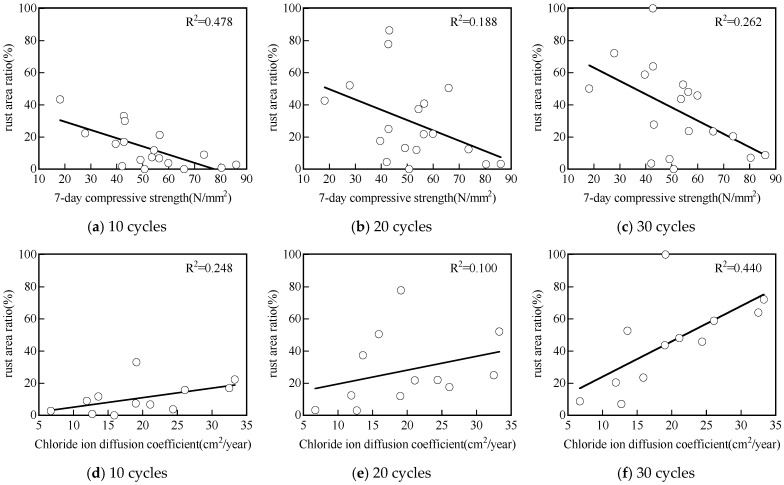
Correlation between corrosion area ratio and 7-day compressive strength (**a**–**c**) and chloride ion diffusion coefficient (**d**–**f**).

Figure 11 illustrates the correlation between the rust area and the 7-day compressive strength, as well as the chloride ion diffusion coefficient. All correlations were poor with R^2^ values less than 0.24 for 10, 20, and 30 drying/wetting cycles. For instance, after 20 drying/wetting cycles, concretes with a compressive strength of 40 MPa exhibited a wide range of rust areas, varying from less than 10% to over 80%. Noticeably, the correlation between rust area and 7-day compressive strength was slightly better after 30 drying/wetting cycles in comparison with 10 and 20 drying/wetting cycles.

The poor correlation between rust area and compressive strength aligns with the poor correlation between the compressive strength and chloride resistance of GP reported by previous studies [6,7,26]. Noushini et al. [6,7] reported that the 28-day average compressive strength did not correlate well with the chloride diffusion coefficient obtained using ASTM C1556 [27]. Similarly, Gluth et al. reported a poor relationship between non-steady-state chloride migration coefficients by NT Build 492 [28] and 28-day compressive strength [25].

Furthermore, the correlations between rust area and chloride ion diffusion coefficient were poor for up to 30 drying/wetting cycles, with low R^2^ values of about 0.39 (Figure 11d–f). This indicates that the corrosion of steel bars in GP is not solely dependent on the chloride ion diffusion in GP mixes, i.e., the initiation phase of the reinforcement corrosion process. Other factors, such as the chloride threshold, moisture transport, or oxygen availability at the steel/concrete interface during the propagation phase are also important and are not captured by standard chloride ions diffusion tests. These factors, which greatly influence active steel bars corrosion, are significantly different in GP materials compared to OPC-based concretes [11,16,29,30]. Depending on the precursor and the activator compositions, both the pore structure and the chemistry of the GP matrix can be highly variable. As a result, assessing GP concrete’s durability in chloride environments based solely on compressive strength and/or chloride diffusion coefficient measurement is not accurate. The new testing method proposed, which also includes the propagation phase of the corrosion process by measuring the rust area of steel bars, can potentially greatly improve the performance-based estimation of GP concrete’s durability.

Conventional concrete with high compressive strength tends to be dense and has low chloride ion permeability. This figure shows that GP has different properties from conventional concrete, as the correlation compressive strength and corrosion area ratio are poor.

### 3.4. Relationship Between the Location of Rust Initiation and Surface Cracks and Their Length Change with Wetting and Drying Cycles

**Figure 12:**
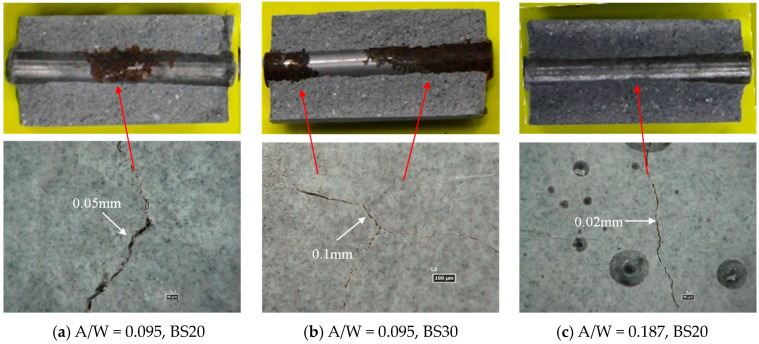
Reinforcing bar element arrangement.

Figure 12 shows an example of the rusting of steel bars and cracks on the specimen’s surface. Overall, based on the location and morphology of the crack, it is difficult to determine whether cracking is due to the pressure from the rust forming or from restrained shrinkage. It is in fact likely to be due to a combination of both effects. In Figure 12a, there is a crack of 0.05 mm width at the center of the specimen in the longitudinal direction, and rust is spreading around it, which suggests that corrosion products formation is likely governing cracking. The mortar in Figure 12b has a higher GGBS replacement ratio than that in Figure 12a, but cracking pattern seems similar to the one observed in Figure 12a, with significant rust volumes observed. Figure 12c (A/W = 0.187) is a case of a higher alkali concentration compared to the mortars presented in Figure 12a,b. No rust is observed, suggesting that cracking is mostly due to restrained shrinkage. The crack width is small (0.02 mm). It is considered that cracks are only surface cracks that do not reach the location of the rebar; hence, no rust is observed on the steel surface at the exact location of the cracks.

The cover thickness of the specimen used in this experiment is 17 mm. Reducing the diameter of the rebar increases the cover thickness, but the evaluation area for rusting is reduced. The simplicity of the experiment was prioritized when selecting the rebar diameter. It is expected that the same results will be obtained under other conditions if there are repeated drying and wetting cycles.

**Figure 13:**
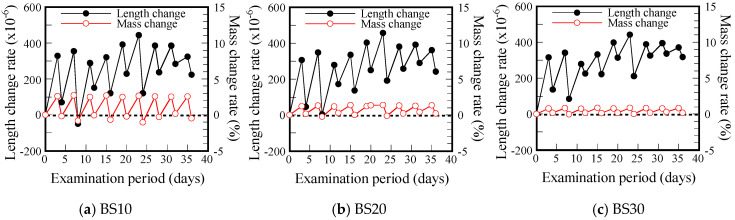
Length and mass change with drying and wetting cycles (in the case of A/W = 0.126)—10 cycles equal 40 days.

Figure 13 shows the length and mass changes of the specimens after the repeated drying and wetting cycles. In the figure, the peak values for both strain and mass change rate are those obtained immediately after removal from the water. The trend of strain change is similar for all the formulations, and the residual strain increases after around three drying/wetting cycles. In other words, cracking is considered to have occurred at this stage, and thereafter the range of variation becomes gradually smaller, indicating that cracks are developing.

**Figure 14:**
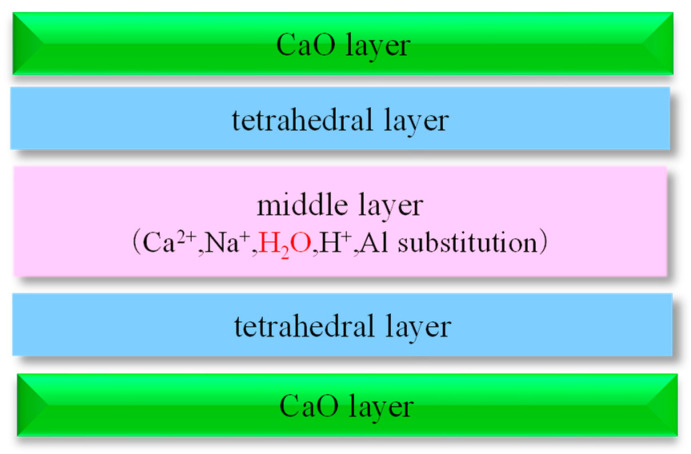
Example of C-A-S-H structure.

On the other hand, the range of variation of the mass loss rate is smaller when there is a higher GGBS replacement ratio. This means that the higher the GGBS substitution ratio, the lower the porosity and likely greater the tortuosity of the pore structure, leading to less water absorption. This is thought to be mainly due to C-A-S-H formed by Ca leached from GGBS. Figure 14 shows an example of the structure of C-A-S-H, which is similar to the structure of smectite, a clay mineral that expands due to water absorption, with a stacked structure consisting of a CaO phase, a tetrahedral layer, and an intermediate layer.

Figure 13 shows that the change in length is the same for all GGBS replacement rates, but the higher the GGBS replacing rate, the less water absorption (i.e., mass change) is observed. This result shows that the GGBS products (C-A-S-H) expand significantly as a result of even a small amount of water absorption. It also suggests that when promoting rust formation under repeated wetting and drying cycles, it is necessary to select experimental conditions that do not cause cracks on the surface of the test specimen, regardless of the BS replacing rate.

## 4. Conclusions

GP, which is produced from alumina–silica powder and alkali–silica solution, solidifies via a condensation polymerization reaction. Compared to conventional cement-solidified products, GP tends to form fine internal voids and has high permeability to substances such as chloride ions. On the other hand, GP has higher adhesion to reinforcing bars than conventional cement based concrete, and a boundary layer may be formed at the interface between the two to inhibit rusting. And it is not clear whether the permeability of chloride ions in the matrix (concrete or mortar) and the corrosiveness of steel bars placed inside GP can be uniquely explained.

In this study, we conducted salt water immersion tests using a 50 mm diameter, 100 mm high cylindrical mortar specimen with a polished rebar placed in the center of its circular cross section to investigate the relationship between the GP composition and curing conditions and the corrosion of the rebar. The length and mass changes of the specimens were also measured, and it was clarified that the cracks observed in the salt water immersion tests were caused by the water-absorption and expansion characteristics of the GP, from which the following conclusions are drawn:

(1) A high GGBS replacement ratio (10–30%) allows us to significantly reduce the rust formation, although GGBS content is not the only GP mix design parameter governing durability and rusting.
(2) The longer the number of days of pre-curing (0 day, 2 days), the better the prevention of rusting. This may be due to the fact that pre-curing promotes the formation of matrix gels (N-A-S-H, C-A-S-H), which leads to a higher density of GP mortar and better adhesion to the steel bars.
(3) No difference in chloride ion diffusion coefficients was observed between the steam curing temperatures of 60 °C and 80 °C. Similarly, no relationship was observed between the rusting conditions and the steam curing temperature.
(4) A higher alkali solution concentration (A/W) tended to reduce rusting.
(5) Poor correlation was observed between the rust area and the mortar’s compressive strength or the chloride ion diffusion coefficient. This indicates that factors such as the chloride threshold, moisture transport, or oxygen availability at the steel/concrete interface during the propagation phase are also important and are not captured by standard chloride ions diffusion tests.
(6) Depending on the precursor and the activator compositions and for a similar compressive strength, both the pore structure and chemistry of the GP matrix can be highly variable. As a result, assessing GP concrete’s durability based solely on compressive strength and/or chloride diffusion coefficient measurement is not accurate. The new testing method proposed, which also includes the propagation phase of the corrosion process by measuring the rust area of steel bars, can potentially greatly improve the performance-based estimation of GP concrete’s durability in chloride environments.
(7) The purpose of this paper was to demonstrate the applicability of a new performance-based test method using cylindrical mortar specimens with embedded rebars. The new performance-based test was successfully validated by showing that it was able to capture the differences in performance between the different mortars tested. The evaluation method introduced here is advantageous in that it is relatively simple and reliable, as it directly observes the development of rust on the rebar.

## Figures and Tables

**Table 1:** Materials used in mortars.

Item	Symbol	Material Names and Properties
Aluminosilicate precursors	FA	Fly ash (JIS Class 1), Density 2.39 g/cm^3^, Specific surface area 5327 cm^2^/g
	GGBS	Ground granulated blast-furnace slag; density 2.92 g/cm^3^; specific surface area 4009 cm^2^/g
Alkali solution	AW	Mixture of water glass, caustic soda and water; density 1.27 g/cm^3^; Si/A 0.613
Fine aggregate	S	Mixed silica sand; density 2.64 g/cm^3^

**Table 2:** Mortar mix design (kg/m^3^). (The blue background indicates A/W = 0.126, which is the basic mixture used in this experiment).

Materials	Mix 1 (BS10)	Mix 2 (BS20)	Mix 3 (BS30)	Mix 4 (BS10)	Mix 5 (BS20)	Mix 6 (BS30)	Mix 7 (BS10)	Mix 8 (BS20)	Mix 9 (BS30)
Fine aggregate	1311.1	1311.1	1311.1	1311.1	1311.1	1311.1	1311.1	1311.1	1311.1
FA	534.8	475.4	416.0	534.8	475.4	416.0	534.8	475.4	416.0
GGBS	73.5	147	230.6	73.5	147	230.6	73.5	147	230.6
Sodium silicate	120.4	120.4	120.4	132.2	132.2	132.2	163.6	163.6	163.6
NaOH pellets	20.6	20.6	20.6	22.6	22.6	22.6	28.0	28.0	28.0
Free water	178.8	178.8	178.8	165.0	165.0	165.0	128.3	128.3	128.3
A/W	0.095			0.126			0.187		
MR ^a^	1.19	1.19	1.19	1.19	1.19	1.19	1.19	1.19	1.19
Na_2_O%/Binder ^b^	6.12	5.98	5.76	6.72	6.56	6.32	8.31	8.12	7.82
Water ^c^/Solid ^d^	0.34	0.33	0.32	0.32	0.31	0.30	0.28	0.27	0.27
GGBS content—Vol.%	10	20	30	10	20	30	10	20	30
GGBS content—Wt.%	12.08	23.62	35.66	12.08	23.62	35.66	12.08	23.62	35.66

^a^: molar ratio of alkaline solution, MR = SiO_2_/Na_2_O. ^b^: Binder = FA + GGBS (in mass). ^c^: Water = free water + water in Sodium silicate. ^d^: Solid in binder = precursor + solid components (Na_2_O and SiO_2_) in sodium silicate + NaOH pellets.

## Data Availability

The original contributions presented in the study are included in the article, further inquiries can be directed to the corresponding author.
